# Analysis of Concussions with Persisting Symptoms Caused by Motor Vehicle Crashes in 136 Vehicle Occupants Shows that Females Are Vulnerable Road Users

**DOI:** 10.1089/neu.2024.0207

**Published:** 2025-06-04

**Authors:** Charles H. Tator, Olivia F.T. Scott, Benjamin S. Elkin, Emma Prentice, Umar Muhammad, Mozhgan Khodadadi, Qixuan Li, Ella Huszti, Maria Carmela Tartaglia

**Affiliations:** ^1^ Division of Neurosurgery, Canadian Concussion Centre, Toronto Western Hospital and University of Toronto, Toronto, Canada.; ^2^ Canadian Concussion Centre, Toronto Western Hospital and University of Toronto, Toronto, Canada.; ^3^ MEA Forensic Engineers & Scientists, Toronto, Canada.; ^4^ Biostatistics Department, University Health Network, Toronto, Canada.; ^5^ Division of Neurology Toronto Western Hospital, Canadian Concussion Centre, University of Toronto, Toronto, Canada.

**Keywords:** concussion, brain injury, motor vehicle crashes, prevention, recovery, sex incidence

## Abstract

At the Canadian Concussion Centre, we treated 136 patients from 2000 to 2020 who sustained concussion plus persisting concussion symptoms (C+PCS) as motor vehicle occupants involved in motor vehicle crashes (MVCs). This center specializes in the treatment of patients with C+PCS. The objective of the present study was to identify strategies for preventing concussion among vehicle occupants involved in MVC. Indeed, this is the first study focused on C+PCS in MVC occupants, and our main purpose was to evaluate the effectiveness of onboard concussion prevention strategies. In this retrospective, consecutive cohort of 136 patients with C+PCS, we examined the patients’ demographic and injury features in relation to the nature of the MVC including speed, direction of impact, and availability, deployment, and effectiveness of onboard occupant safety measures including seatbelts, head restraints, and airbags. The most frequent combination of factors was a belted female driver of an automobile struck from behind by another automobile. Surprisingly, the entire patient cohort comprised more females (69.1%) than males (30.9%), and rear-end collision was the most common type in females. Most injured occupants of both sexes were wearing seatbelts, but only a minority of the crashes caused airbag deployment. The seven most common symptoms were headache (84.6%), anxiety (72.8%), sensitivity to light (70.6%), memory problems (69.9%), sensitivity to noise (66.2%), irritability (56.6%), and depression (55.9%). Whiplash was a frequent associated injury in both sexes. Complete recovery from C+PCS was rare, and most patients with known follow-up continued to suffer from persisting symptoms for months to years. The median symptom duration for all 136 patients was 30.0 months (interquartile range: 16.8–56.0 months). Based on these findings, we conclude that females are indeed vulnerable road users with respect to C+PCS, and our literature search showed that there had been some previous evidence of increased injury risk of other injuries in female occupants. We recommend that additional prevention strategies are required to reduce the post-crash acceleration–deceleration “bobble-head” movement of the head on trunk causing both concussion and whiplash as has been accomplished in auto racing. Also, these prevention measures must be investigated in crash studies that include low-to-high speed rear-end collisions using anthropometrically appropriate models of male and female occupants reflecting the range of sizes of both sexes. There is a need for more concussion brain injury prevention research focusing on the vulnerability of female occupants, which has not been sufficiently addressed even though the deficiency was identified many years ago. The sex inequity of current onboard motor vehicle concussion brain injury prevention measures especially with respect to females should be addressed by governments and the automobile and insurance industries.

## Introduction

Most concussed patients fully recover from concussions within about one month, and those who do not recover within this time are considered to have persisting concussion symptoms (PCS), which we designate as concussion plus PCS (C+PCS). Previously, this condition was termed post-concussion syndrome.^1^ The Canadian Concussion Centre (CCC) specializes in the treatment of patients with C+PCS, and we found that motor vehicle crash (MVC) was the cause in many patients we treated who suffered from C+CPS.^2^ The objective of the present study was to identify strategies for preventing concussion among occupants of vehicles involved in MVC. Indeed, this is the first study focused on C+PCS in MVC occupants, and one of our main purposes was to evaluate the effectiveness of on-board injury prevention strategies to prevent C+CPS. Al-Balbissi in 2003^3^ performed a detailed analysis of sex as a factor in the epidemiology of MVC in many countries and showed “significantly higher accident rates for male drivers compared with female drivers,” and most subsequent analyses have concurred. However, there is also evidence that a higher percentage of belted female occupants involved in MVC sustain more injuries of specific types than males, which has led many to question the sex equality of current injury prevention measures.^4^ However, there have been no previous published studies comparing concussion rates after MVC in male and female occupants.

In the past 20 years, there has been a remarkable reduction in the number of fatalities from MVC in many countries including Canada^5,6^ due to the widespread adoption of seatbelts, head restraints, and airbags. However, there has also been mounting evidence that females are at greater risk than males with respect to specific injuries incurred in MVC including some types of brain injury and whiplash^4^ and that vehicle design may be the problem.^7^ Indeed, one study found that in general, “females were at greater risk of more severe injuries than males for most types of injuries” sustained in MVC but only frontal mechanisms were examined, and there was no specific analysis of concussions.^8^ Another recent study evaluating sex differences in MVC included an extensive analysis of survivors, but the inclusion criteria was hospitalization for at least 3 days which would have excluded the majority of concussed patients, and there was no specific analysis of concussions.^9^ However, they found several significant sex differences in the incidence of major injuries including spinal injuries.

One of the major deficiencies in injury analysis, in general, is the inequality of available crash test forms for women compared with men. This is considered to be a major deficiency with respect to vehicle protection studies for women. Also, to our knowledge, no specific bioengineering analysis of concussion has been done in MVC studies of all types, either clinical, simulated virtual or with crash-test automobiles to compare the risk and outcome from concussions in males and females due to clinically relevant forces experienced in the various types of MVC such as those identified in the present study. The present study is the first to analyze sex differences in the occurrence of C+PCS related to front, rear, side, and other crash types and to assess prevention of occupant concussions by current onboard protective equipment including seatbelts, head restraints, and airbags. In our recent study of 600 patients with C+PCS, MVC was among the four most common causes of C+PCS, and most were occupants.^2^ We hypothesized that a detailed analysis of concussions sustained by occupants in MVC will reveal information useful for enhancing the treatment and prevention of concussions, especially among female occupants.

## Methods

### Study design and participants

This retrospective cohort of 136 consecutive patients of ages 15–81 with C+PCS after an MVC was referred to one of the authors (C.H.T.) at the CCC, Toronto Western Hospital, University Health Network (UHN), and University of Toronto between January 2000 and May 2020. This center specializes in the management and treatment of C+PCS and serves a patient population from across the province of Ontario, both urban and rural. All patients were examined by physicians trained in concussion. The study was approved by the UHN Research Ethics Board, and all patients who completed the final follow-up questionnaire signed consent. The treatment offered for PCS was multidisciplinary, individualized, and symptom-oriented for managing headaches, mental health issues, dizziness, sleep disorder, and other symptoms with an emphasis on early return to physical and cognitive activity and respect for thresholds at which symptom exacerbation occurred. The CCC focuses on long-term management of C+PCS through a policy of providing long-term follow-up through repeated visits and periodic questionnaires to assess the longitudinal effects of concussion. To accomplish this, all patients were specifically directed to return for at least one or more follow-up visits, and after completion of follow-up office visits, patients were requested to complete follow-up questionnaires mailed to them approximately every 18–24 months until they had completely recovered from C+PCS.

### Inclusion/exclusion criteria

All patients sustained C+PCS as a motor vehicle occupant involved in an MVC with at least one symptom lasting at least one month (see definition of PCS, below).

### Data collection

Data were collected through a review of clinical records, and periodic follow-up CCC questionnaires sent to the patients. The clinical records examined included the physicians’ concussion clinic notes, information from the referring physician or other healthcare professionals, police reports, photographs of the injured patient and crashed vehicles, collision center reports, legal documents shared with the examining physician, and patient self-reports including the questionnaires sent to the patients (see below). The mechanisms of the MVC and the symptoms were documented based on the above. In 2021 and 2022, we sent a final follow-up questionnaire and made telephone calls to all 136 patients to ask additional MVC-related questions to which 37 patients responded.

### Study variables and definitions

*Demographics:* sex, age.

*Concussion:* Defined according to the Fifth International Conference on Concussion in Sport^10^

*Index Concussion*: The index concussion was defined as the concussion resulting from the MVC which brought the patient to the CCC for consultation.

*Time from index concussion to first CCC visit:* The mean time from index concussion to first clinic visit was 11.9 (standard deviation [SD] = 16.9) months.

*Persisting Concussion Symptoms*: The definition of PCS was as follows: 1) at least one persisting symptom; 2) persistence of symptom(s) for at least one month; and 3) absence of evidence of more severe traumatic brain injury such as intracranial hemorrhage, cerebral contusion, or clinical neurological defects such as limb weakness, or other lesions seen on magnetic resonance imaging or computerized tomographic scans based on our reported criteria.^11^ Thus, the diagnosis of concussion was made clinically. All patients in this cohort were referred to and examined by one of the authors (CHT) and diagnosed with PCS. PCS symptoms were categorized as somatic, cognitive, or neuropsychiatric (affective).

*Recovery from C+PCS:* We used a new method for assessing recovery from concussion, which involved three categories of recovery all measured in months from the time of the index concussion as reported previously:^2^

1. Complete Recovery: Defined as absence of all PCS as reported by the patient.
2. Incomplete Recovery: Patients with at least one follow-up visit or one returned questionnaire who reported persisting symptoms at their most recent visit or questionnaire.
3. Unknown Recovery: Patients with PCS who had only an initial visit, no follow-up visit, and failed to return any questionnaires.

*Questionnaires:* Questionnaires were sent every 1–2 years to patients who had not recovered. In addition, the 136 patients in the present study were sent an additional “final” questionnaire in 2021 and 2022 with specific questions about recovery. The final questionnaire asked for additional specific information related to the MVC that may not have been asked previously and for purposes of obtaining MVC details important for prevention.

*Symptom Duration:* Defined as the duration in months during which patients suffered symptoms from their index concussion.

*Follow-Up Duration:* efined as the time in months patients were followed by visits or questionnaires after their index concussion. For patients with incomplete recovery, follow-up duration was equal to symptom duration. For patients with complete recovery, follow-up duration was either equal to symptom duration (when patients reported complete recovery at their most recent follow-up) or greater than symptom duration (when patients completely recovered prior to their latest follow-up).

*Collision Information:* The type of MVC was categorized as rear-end, frontal, lateral including T-bone, or other, which included sideswipe, single car, multiple collisions, rollover, and unknown. The occupant’s position in the vehicle included driver or passenger and front or rear seat. All of the patients’ signs and symptoms were recorded including any loss of consciousness. In those without loss of consciousness, we recorded information about what objects in the vehicle that the occupant’s head may have struck such as the windows, pillars between the windows, deployed airbags, dashboard, and steering wheel and in what sequence.

*Vehicle Safety Systems:* We recorded seatbelt usage, airbag deployment, and position of the head restraint.

*Additional Variables from the Final Follow-Up Questionnaire n* = 37: This questionnaire included the following variables: type of vehicle; vehicle model year; maximum allowed speed of the road on which the crash occurred; actual speed of the vehicle or vehicles involved in the collision, or if the patient’s vehicle had fully stopped; time of day; did the patient see the vehicle with which their vehicle crashed prior to the collision (if applicable); did the patient brace for the impact; was it a single vehicle collision; was the vehicle drivable after the crash; patient’s head position; patient’s head restraint position and did the patient’s head hit an object within the vehicle and what that object was; did the patient also sustain whiplash, low back, or shoulder injuries, or other injuries; and the extent to which the concussion impacted their life.

### Statistical analysis

Demographic characteristics of the patients were summarized using the median ([interquartile range [IQR]) for continuous variables and number (%) for categorical ones. T-tests, Wilcoxon nonparametric, Chi-square, and Fisher’s exact tests were used for comparisons among different groups. The association of various risk factors (sex, age, type of MVC, and occupant position in the vehicle) with the total number of PCS was assessed by using univariable and multivariable Poisson regression models. We also tested the interaction between sex and type of MVC in the Poisson models. In a sensitivity analysis, we repeated the Poisson models after excluding the patients with less than three symptoms. A *p* value of <0.05 was considered significant. All analyses were performed in R.4.3.1.

## Results

### Patient and collision characteristics

The patient and collision characteristics are presented in Table 1. Of the 136 included patients, 94 (69.1%) were female and 42 (30.9%) were male (*p* < 0.001 for difference from equal 50% male/female). The average age of all participants was 41.4 (SD = 13.9) years, with no differences in mean age between females and males (42.0, SD = 12.51 and 40.0, SD = 16.56, respectively).

**Table 1.** Patient, Collision/Injury, and Recovery Variables (*N* **=** 136 C+PCS Patients)

Variables	*n* (%) unless otherwise specified	Missing *n* (%)
1. Patient variables		
Sex		
Female	94 (69.1)	0.0
Male	42 (30.9)	
Age mean (SD)		
All patients	41.4 (13.9)	0.0
Female	42.0 (12.5)	0.0
Male	40.0 (16.6)	0.0
Type of MVC		
Rear	69 (50.7)	0.0
Frontal	15 (11.0)	
Lateral	29 (21.3)	
Other^a^	23 (16.9)	
Occupant position in the vehicle		
Driver	95 (71.4)	3 (2.2)
Passenger	38 (28.6)	
2. Collision/Injury variables		
Loss of consciousness		
Yes	25 (18.4)	0.0
No	111 (81.6)	
Seatbelt use *n* = 93		
Yes	87 (93.5)	43 (31.6)
No	6 (6.5)	
Airbag deployment *n* = 82		
Yes	24 (29.3)	54 (39.7)
No	58 (70.7)	
3. Recovery variables		
Recovery group		
Complete recovery	3 (2.2)	
Incomplete recovery	113 (83.1)	
Unknown recovery	20 (14.7)	
Follow-up duration in months (median [IQR])	30.0 [16.8, 56.5]	
Symptom duration in months (median [IQR])	30.0 [16.8, 56.0]	
Number of persisting symptoms (mean [SD])		
Somatic symptom total	6.96 (4.2)	
Cognitive symptom total	2.15 (1.7)	
Neuropsychiatric symptom total	4.28 (2.9)	
Total persisting symptoms	13.39 (7.9)	

^a^ Other includes sideswipe, single car collision, multiple collisions, and unlisted.

IQR, interquartile range; MVC, motor vehicle collision; PCS, persisting concussion symptoms; SD, standard deviation.

**FIG. 1.**
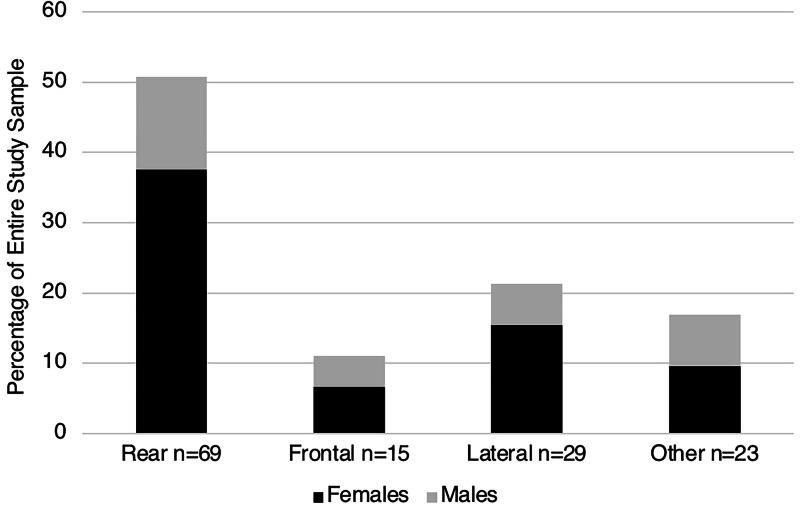
Rear-end collisions were the most common type of collision *p* < 0.001, and females who sustained their concussion in a rear-end collision or lateral collision were the most prevalent group and more females were injured in rear and lateral collisions than males. The “Other” group included sideswipe, single car, rollover, multiple collisions, and unknown. Of the 69 rear-end collisions, 51 (73.9%) were in females and 18 (26.1%) were in males.

Table 1 also shows that the most common type of MVC was rear-end (50.7%) followed by lateral (21.3%), other (16.9%), and frontal (11.0%) (*p* < 0.001 for difference from equal 25% each). When stratified by sex, we found that females concussed in a rear-end collision were the most prevalent group in our sample, comprising 37.5% of our overall sample (Fig. 1). With respect to position in the vehicle, 71.4% were driving at the time of the collision, while 28.6% were passengers. As a result of the collision, 18.4% experienced loss of consciousness (LOC) (Table 1). Table 2 lists the incidence of 53 symptom types experienced by the 136 patients. There were no significant differences in symptoms between the four MVC collision types other than for LOC, which was most common in the “other” category of collisions (43.5%) followed by frontal (33.3%), lateral (17.2%), and rear-end collisions (7.2%) (*p* = 0.001). The “other” category included sideswipe, single car, multiple collisions, rollover, and unknown.

**Table 2.** Number and Proportion of Patients (*n* = 136) Experiencing 53 Self-Reported Persisting Concussion Symptoms (A. Somatic *n* = 26, B. Cognitive *n* = 12, C. Neuropsychiatric *n* = 15) in Order of Decreasing Symptom Prevalence^a^

Self-reported symptoms (*n* **=** 53)	Number of patients reporting the symptom	Proportion of patients reporting the symptom
A. Somatic symptoms (*n* = 26)		
Headache	115	84.6
Sensitivity to light	96	70.6
Sensitivity to noise	90	66.2
Fatigue	74	54.4
Balance problems	71	52.2
Neck pain	68	50
Pressure in the head	58	42.6
Dizziness	54	39.7
Nausea	46	33.8
Vertigo	42	30.9
Tinnitus	37	27.2
Numbness	35	25.7
Blurred vision	26	19.1
Light-headedness	24	17.6
Vision changes	22	16.2
Increased sensitivity to alcohol	15	11
Loss of appetite	12	8.8
Dazed	11	8.1
Slurred speech	11	8.1
Vomiting	11	8.1
Double vision	7	5.1
Speech problems	7	5.1
Stomach ache	6	4.4
Seizures	4	2.9
Clumsiness	2	1.5
Staring vacantly	2	1.5
B. Cognitive symptoms (*n* = 12)		
Memory problems	95	69.9
Concentration difficulties	74	54.4
Mental fogginess	39	28.7
Feeling slowed down	34	25
Confusion	29	21.3
Disorientation	11	8.1
Attention difficulties	5	3.7
Thinking time increased	2	1.5
Distracted easily	1	0.7
Learning difficulties	1	0.7
Problem-solving difficulties	1	0.7
Response speed slowed	1	0.7
C. Neuropsychiatric symptoms (*n* = 15)		
Anxiety	99	72.8
Irritability	77	56.6
Depression	76	55.9
Sleeping too little	64	47.1
Increased emotionality	51	37.5
Sadness	44	32.4
Frustrated	42	30.9
Insomnia	32	23.5
Did not feel right	29	21.3
Panic attacks	27	19.9
Personality changes	20	14.7
Sleeping too much	11	8.1
Aggression	7	5.1
Apathy	2	1.5
Restlessness	1	0.7

^a^ Wherever possible, symptoms were recorded in language as close as possible to the patients’ description of the symptom.

### Vehicle safety systems

**FIG. 2.**
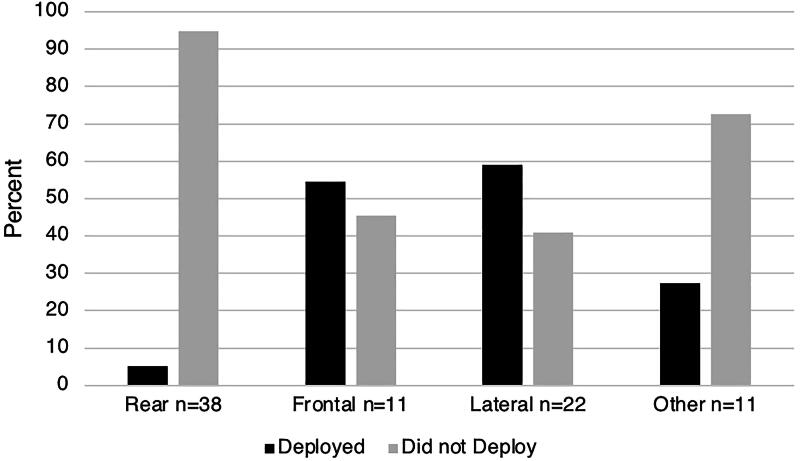
Airbag deployment almost never occurred in rear-end collisions, but airbags deployed in more than half of front end and side collisions. *p* < 0.001. The “Other” group included sideswipe, single car, rollover, multiple collisions, and unknown.

Data on seatbelt usage and airbag deployment were available from patients’ charts or questionnaires for 92 (67.6%) and 82 (60.3%) patients, respectively (Table 1). We found that 87 out of 93 (93.5%) with available data were wearing a seatbelt at the time of collision and that seatbelt usage did not differ significantly between the four types of MVCs. In contrast, the overall airbag deployment rate in crashes was only 24 out of 82 (29.3%) and differed significantly between the four types of MVCs (*p* < 0.001) (Fig. 2): airbags deployed in only 5% of rear-end collisions, whereas deployment occurred in more than 50% of frontal and lateral collisions and 27.3% in the “other” types of collision category.

### Recovery from concussion plus persisting concussion symptoms

Complete recovery from C+PCS caused by MVCs was low, with only 3 (2.2%) patients achieving complete recovery from all PCS. Most patients had incomplete recovery (83.1%), and recovery information was unknown in 14.7% (Table 1). Overall, the median follow-up and symptom duration were both 30 months (follow-up duration: 30.0 months, IQR = 16.8–56.5; symptom duration: 30.0 months, IQR = 16.8–56.0). Patients with complete recovery had a shorter symptom duration (*n* = 3; median = 10 months) compared to patients with known incomplete recovery (*n* = 113; median = 32 months), and patients with unknown recovery had the shortest recorded symptom duration (*n* = 20; median = 9 months) due to lack of compliance with follow-up clinic visits and/or lack of completion of questionnaires.

The authors are aware of the difficulty of accurately assessing recovery when patient compliance creates data gaps. The new system that we recently reported represents an attempt to utilize as much data as possible to characterize recovery in terms of what is known about the duration of suffering from symptoms in patients with unknown recovery.^2^

In general, patients with C+PCS from an MVC were highly symptomatic, experiencing an average of 13.4 (SD = 7.9) PCS distributed among the three symptom categories: somatic were most common (mean = 7.0, SD = 4.2), followed by neuropsychiatric (mean = 4.28, SD = 2.9), then cognitive (mean = 2.2, SD = 1.7). The four types of MVCs did not differ significantly with respect to concussion recovery and symptom variables (Tables 1 and 2). One patient had only one symptom, three patients had only two symptoms, with the remainder having three or more symptoms. The number and prevalence of each of the specific 53 reported symptoms in Table 2 included somatic (*n* = 26), cognitive (*n* = 12), and neuropsychiatric (*n* = 15) categories. The three most prevalent somatic symptoms were headache (*n* = 115, 84.6%), sensitivity to light (*n* = 96, 70.6%), and sensitivity to noise (*n* = 90, 66.2%), whereas the three most common cognitive symptoms were memory problems (*n* = 95, 69.9%), concentration difficulties (*n* = 74, 54.4%), and mental fogginess (*n* = 39, 28.7%), and in the neuropsychiatric category, the three most prevalent symptoms were anxiety (*n* = 99, 72.8%), irritability, (*n* = 77, 56.6%), and depression (*n* = 76, 55.9%).

### Univariable and multivariable Poisson models for total number of persisting symptoms

In univariable analyses, sex, type of MVC, and occupant position in the vehicle were significantly associated with total number of persisting symptoms (Table 3). Females had a 14% higher risk of having a greater number of PCS when compared to males (risk ration [RR] = 1.14; 95% confidence interval [95% CI] [1.03, 1.26], *p* = 0.011). Patients concussed in a rear-end, or lateral collision had a 27% and 36% higher risk of a greater number of PCS, respectively, when compared to frontal collisions (rear-end: RR = 1.27; 95% CI [1.07, 1.49], *p* < 0.01; lateral: RR = 1.36; 95% CI [1.34, 1.62], *p* < 0.001). Drivers were 12% more likely to have a higher number of PCS than passengers (RR = 1.12; 95% CI [1.01, 1.25], *p* = 0.03). Finally, there was no association between the total number of persisting symptoms and age or “other” types of collisions compared to front-end collisions.

**Table 3.** Univariable and Multivariable Poisson Models for Associations with Total Number of Persisting Concussion Symptoms. (*n* = 133)

Variables	Univariable RR (95% CI)	*p* value	Multivariable^a^ RR (95% CI)	*p* value
Sex				
Male	Refr.			
Female	1.14 (1.03–1.26)	0.011	1.09 (0.98–1.21)	0.102
Age	1.003 (0.99–1.01)	0.105	1.001 (0.99–1.01)	0.508
Type of MVC				
Frontal	Refr.			
Rear	1.27 (1.07–1.49)	<0.01	1.24 (1.05–1.46)	0.011
Lateral	1.36 (1.13–1.62)	<0.001	1.33 (1.11–1.59)	<0.01
Other^b^	0.91 (0.75–1.11)	0.36	0.92 (0.75–1.12)	0.40
Occupant position in the vehicle				
Passenger	Refr.			
Driver	1.12 (1.01–1.25)	0.03	1.09 (0.98–1.22)	0.13

^a^ All variables (sex, age, type of MVC, and occupant position) were included in the multivariable model.

^b^ Other includes sideswipe, single car collision, multiple collisions, and unlisted.

CI, confidence interval; MVC, motor vehicle collision; RR, risk ratio.

In a multivariable model (Table 3) that included all variables, both rear-end and lateral collisions remained significantly associated with total number of PCS when compared to patients who were concussed in a frontal collision (rear-end: RR = 1.24; 95% CI (1.05, 1.46), *p* = 0.01; lateral: RR = 1.33; 95% CI (1.11, 1.59), *p* < 0.01).

There was no significant interaction between sex and type of MVC (*p* values: 0.3; 0.3; 0.1), and therefore, the association between type of MVC and total number of PCS was similar for males and females.

In a sensitivity analysis excluding the four patients who had < 3 PCS symptoms, the results remained similar. In the multivariable model, females had a 12% higher risk of having a greater number of PCS when compared to males (RR = 1.12; 95% CI [1.01, 1.25], *p* = 0.035), while the associations of the other types of collisions with total number of PCS when compared to frontal collision became (rear-end RR = 1.14; 95% CI [0.96, 1.35], *p* = 0.13 and lateral RR = 1.22; 95% CI [1.02, 1.46], *p* = 0.03).

### Questionnaire data

Table 4 shows the features of the 37 (27.2%) patients of the 136 study patients who completed the final questionnaire, and Table 5 compares these 37 patients with the 99 who did not complete the questionnaire. Table 4 shows that most patients were driving a sedan (63.6%), were on a main road, and if they were in motion their speed at the time of the collision was 50–60 km/h in about 50% of cases. In most cases, their collisions were in the afternoon (57.1%; 12:01 p.m.–6:00 p.m.) (Table 4). Eighteen (48.6%) patients were in motion at the time of the collision, and 19 (51.4%) were completely stopped. For patients in motion at the time of the collision and whose vehicle speed was known (*n* = 14), the average speed was 42 km/h (SD = 26.9). When a second vehicle was involved in the collision and its speed was known (*n* = 24), its average speed was 60.73 km/h (SD = 31.67). Importantly, for rear-end collision analysis, 17 (45.9%) of the respondents were completely stopped when struck from behind by vehicles traveling at speeds up to 120 km/h, with an average closing speed of 55.19 km/h (SD = 27.36) (*n* = 13).

**Table 4.** Final Questionnaire Data (*n* = 37 Patients; Some Subjects Did Not Answer All Questions)

Variable	*n* (%) unless otherwise specified	Missing *n* (%)
Sex		
Female	29 (78.4)	0 (0.0)
Male	8 (21.6)	
Type of MVC		
Rear	20 (54.1)	0 (0.0)
Frontal	1 (2.7)	
Lateral	12 (32.4)	
Other	4 (10.8)	
Type of vehicle		
Sedan	21 (63.6)	4 (10.8)
SUV	5 (15.2)	
Hatchback	3 (9.1)	
Pickup	3 (9.1)	
Minivan	1 (3.0)	
Vehicle model year		
2000–2005	5	6 (16.2)
2006–2010	11	
2011–2015	10	
2016–2020	5	
Type of road		
Street	7 (19.4)	1 (2.7)
Main road	20 (55.6)	
High-speed road	3 (8.3)	
Highway	6 (16.7)	
Time of day		
Morning	6 (21.4)	9 (24.3)
Afternoon	16 (57.1)	
Evening/Night	6 (21.4)	
Head position		
Looking straight ahead	20 (60.6)	4 (10.8)
Turned to the side	13 (39.4)	
Headrest position		
Up	18 (72.0)	12 (32.4)
Down	7 (28.0)	
Was the patient’s vehicle stopped?		
Yes	19 (51.4)	
No	18 (48.6)	0 (0.0)
Speed of the patient’s vehicle (if in motion) in km/h mean (SD)	42 (26.9)	4 (22.2)
Did the patient brace for the impact?		
Yes	8 (25.0)	
No	24 (75.0)	5 (13.5)
Did the patient’s head hit an object in front?		
Yes	12 (42.9)	9 (24.3)
No	16 (57.1)	
What did the patient’s head hit in front (where applicable)		
Steering wheel	5 (41.7)	0 (0.0)
Other object	7 (58.3)	
Did the patient’s head hit an object behind?		
Yes	22 (75.9)	
No	7 (24.1)	8 (21.6)
What did the patient’s head hit behind? (where applicable)		
Head restraint	17 (85.0)	17 (45.9)
Other object	3 (15.0)	
Was the vehicle drivable?		
Yes	14 (38.9)	1 (2.7)
No	22 (61.1)	
Did the patient also sustain a whiplash injury?		
Yes	24 (68.6)	2 (5.4)
No	11 (31.4)	
Whiplash stratified by type of MVC		
Rear	18/20 (90.0)	0 (0.0)
Frontal	0/1 (0.0)	0 (0.0)
Lateral	5/10 (50.0)	2 (16.7)
Other	1/4 (25.0)	0 (0.0)
Did the patient also sustain a lower back injury		
Yes	17 (50.0)	3 (8.1)
No	17 (50.0)	
Did the patient also sustain a shoulder injury		
Yes	14 (43.8)	5 (13.5)
No	18 (56.2)	
To what extent has the patient’s concussion(s) impacted their life?		
Not at all	1 (2.8)	
A little	1 (2.8)	1 (2.7)
A moderate amount	5 (13.9)	
Very much	11 (30.6)	
An extreme amount	18 (50.0)	
Was it a single car collision?		
Yes	3 (8.1)	0 (0.0)
No	34 (91.9)	
For collisions involving another vehicle (*n* = 34)		
Speed of the other vehicle in km/h (mean [SD])	60.73 (31.67)	10 (29.4)
Did the patient see the other car coming		
Yes	10 (29.4)	1 (0.03)
No	23 (67.6)	
Preexisting conditions (*n* = 37)		
Migraines		
Yes	7 (18.9)	2 (5.4)
No	28 (75.7)	
Depression or anxiety		
Yes	15 (40.5)	1 (2.7)
No	21 (56.8)	
Vertigo or dizziness		
Yes	5 (13.5)	1 (2.7)
No	31 (83.8)	
Attention-deficit disorder (ADD)		
Yes	3 (8.1)	2 (5.4)
No	32 (86.5)	
Learning disability		
Yes	1 (2.7)	3 (8.1)
No	33 (89.2)	
Brain disorders such as stroke, tumor, or seizure		
Yes	1 (2.7)	2 (5.4)
No	34 (91.9)	

km/h, kilometers per hour; MVC, motor vehicle collision; SD, standard deviation.

**Table 5.** Patient and Collision Variables Based on Completion (*n* **=** 37) Versus Noncompletion (*n* **=** 99) of the Final Questionnaire

Variable	Questionnaire NOT completed *n* (%) unless otherwise specified	Questionnaire completed *n* (%) unless otherwise specified	*p* value	Missing
*n*	99	37	—	—
Sex (%)				
Female	65 (65.7)	29 (78.4)	0.222	0.0
Male	34 (34.3)	8 (21.6)		
Age (mean [SD])	39.94 (13.75)	45.27 (13.5)	0.045*	0.0
Recovery group				
Complete	2 (2.0)	1 (2.7)	0.054	0.0
Incomplete	78 (78.8)	35 (94.6)		
Unknown	19 (19.2)	1 (2.7)		
Follow-up duration in months (median [IQR])	25.0 [14.5, 38.0]	65.0 [33.0, 98.0]	<0.001*	0.0
Symptom duration in months (median [IQR])	25.0 [14.5, 38.0]	62.0 [33.0, 98.0]	<0.001*	0.0
Type of MVC				
Rear	49 (49.5)	20 (54.1)	0.061	0.0
Front	14 (14.1)	1 (2.7)		
Side	17 (17.2)	12 (32.4)		
Other	19 (19.2)	4 (10.8)		
Occupant position in the vehicle				
Driver	66 (68.8)	29 (78.4)	0.375	3 (2.2)
Passenger	30 (31.2)	8 (21.6)		
Persisting symptoms (median [IQR])				
Somatic symptom total	6.0 [4.0, 8.0]	8.0 [5.0, 13.0]	0.002*	0.0
Cognitive symptom total	2.0 [1.0, 2.0]	4.0 [2.0, 5.0]	<0.001*	0.0
Neuropsychiatric symptom total	3.0 [2.0, 5.0]	7.0 [3.0, 9.0]	0.001*	0.0
Total persisting symptoms	11.0 [8.0, 14.0]	20.0 [10.0, 25.0]	<0.001*	0.0
Loss of consciousness				
Yes	20 (20.2)	5 (13.5)	0.517	0.0
No	79 (79.8)	32 (86.5)		
Airbag deployment				
Yes	14 (31.1)	10 (27.0)	0.872	54 (39.7)
No	31 (68.9)	27 (73.0)		
Seatbelt use				
Yes	53 (94.6)	34 (91.9)	0.922	43 (31.6)
No	3 (5.4)	3 (8.1)		

* *p* value significant at *p* < 0.05.

IQR, interquartile range; MVC, motor vehicle collision; SD, standard deviation.

Most of the 37 patients who answered the final questionnaire reported that they were looking straight ahead at the time of the collision (60.6%), and most had the head restraint positioned in the “up” rather than “down” (72.0%) position (Table 4). With respect to reported contact between the patients’ head and structures in the vehicle, 75.9% reported hitting the back of their head on a structure inside the vehicle, most commonly the head restraint (85.0%), and 42.9% reported striking the front of their head during the collision, most commonly against the steering wheel (41.7%), and in most instances, after striking their head on the seat restraint, especially in rear-end collisions. Of the seven patients who reported hitting both the front and back of their head on an object in the vehicle, all were female. In addition to concussion, many patients sustained other injuries during the MVC including whiplash, lower back injuries, and shoulder injuries. Whiplash was the most common concurrent injury (68.6%), followed by lower back injuries (50.0%) and shoulder injuries (43.8%). Patients were asked to what extent the concussion impacted their life and 50.0% indicated “an extreme amount,” 30.6% “very much,” 13.9% “a moderate amount,” and 2.8% “a little” or “not at all” (Table 4).

The final questionnaire also included questions about preexisting conditions (Table 4). Notably, of the 37 patients who completed the questionnaire, 15 (40.5%) reported having depression or anxiety, seven (18.9%) reported having migraines, and five (13.5%) reported having vertigo or dizziness before their concussion. With respect to the other preexisting conditions included in the questionnaire (attention deficit disorder, learning disability, and brain disorders such as stroke, tumor, or seizure), for each of these conditions, <10% of patients reported having a history prior to their concussion. The patients who completed the questionnaire were significantly older, had significantly longer follow-up and symptom durations, and were significantly more symptomatic compared to patients who did not complete the questionnaire (Table 5).

## Discussion

Blunt force trauma is the leading cause of death for motor vehicle occupants with head injury being the most common lethal event.^12^ MVC is a leading cause of all types of brain injury^4^ including concussion in many countries. Although most concussed patients recover within one month, many will have long-term deficits that interfere with return to work, school, or play and remain symptomatic from C+PCS for months or years.^1^ Many require prolonged multidisciplinary care at great expense. Although there has been great effort to prevent severe brain injury in motor vehicle occupants, less attention has been directed to preventing concussion among motor vehicle occupants. Indeed, concussion after MVC has emerged as a major public health problem in many countries due to its high incidence and prolonged disability, and recently, there has been greater effort to understand the mechanisms.^13,14^ For example, it has been shown that seatbelts alone are insufficient to prevent these concussions.^15^ Although more males are killed in MVC, female occupants are more likely to sustain nonfatal injuries like concussion, although the reasons are unclear. Indeed, many years ago, this led to a call for greater use of anthropometrically configured female models for crash tests.^4^ Although considerable effort has been made to reduce deaths with prevention strategies such as seatbelts and airbags, our recently published study^2^ and the present study show that MVCs continue to cause many concussions among occupants involved in MVC. CCC data since intake for the present study ended in May 2020 indicate that the problem has not changed. Thus, additional prevention strategies are required.

We are not aware of any previous analysis of MVC occupants with C+PCS in relation to variables such as rear-end versus frontal collisions, vehicle speed, seating location, and other passenger safety measures such as airbags. Although rear-end collision is the highest risk for whiplash,^16^ there are no previous detailed analyses about the type of collision, direction of force, or speed of the vehicles involved on the incidence of concussion alone or C+PCS. The main purpose of this retrospective study of a consecutive cohort of 136 concussed MVC occupants with PCS was to determine the crash factors responsible for C+PCS in relation to occupant sex, age, and seating location to identify strategies for improving concussion prevention. We were alerted to the importance of MVC as a cause of C+PCS, especially in women through our previous study of 600 patients with C+PCS, which included MVC as one of the four most common causes of C+CPS in our data bank. We reported that “the MVC group had the highest percentage of females (75.7%), the oldest participants (median: 40.0 [IQR: 30.5–49.0] years), the most symptoms (median: 11.0 [IQR: 8.5–15.0]), and the longest symptom duration (median: 28.0 [IQR: 12.0–56.00] months).”^2^

Despite male drivers being more likely than female drivers to sustain MVC in Ontario,^17^ in the present study of concussed occupants of MVC, we found a greater number of females with C+PCS than males in all types of MVC, suggesting that female vehicle occupants are at greater risk for developing C+PCS following MVC. In contrast, a number of studies of concussion in sports and recreation including our own have found a greater number of males than females with C+ PCS.^18^ In 2011, Bose et al.^4^ strongly advocated more efforts “to address the sex-specific disparity” of MVC injuries that they had found and stated that “health policies and vehicle regulations must focus on effective safety designs specifically tailored toward the female population for equity in injury reduction.” In the present study population of C+PCS in MVC from 2000 to 2020, we found that concussed female occupants were overrepresented especially as occupants in rear-end collisions. In contrast, current occupant safety systems have focused on frontal crush zones to dissipate impact force on cabin occupants, seatbelts, head restraints, and airbags, but our data indicate that females, especially, are not adequately protected from concussions, and that this is most evident in rear-end collisions, even though rear-end collisions are not the most common type of collision on Ontario’s roads and indeed there are far fewer rear-end collisions than single car collisions, which are the most common cause of fatalities, for example.^5^

Hopefully, this new information will stimulate action from governments, highway safety organizations, automobile manufacturers, and insurers. The increased risk to female occupants for C+CPS that we found in the present study adds to a previous study that showed that female vehicle occupants were subject to a greater risk of sustaining concussions in frontal car collisions than males and in “perhaps other crash configurations.”^13^ However, the present study is the first to identify an overrepresentation of concussed females with persisting symptoms after rear-end collisions and to show that rear-end collisions were the most common mechanism in which female occupants were injured. Certainly, our findings indicate a need for further research into these mechanisms and potential prevention strategies. In our data, the typical victim was a belted female driver, often completely stopped in traffic, and then struck from behind by another vehicle. At the moment of the rear-end collision, victims often reported “the bobble-head effect” of their head initially moving backward and striking the head restraint and then recoiling forward, often striking other parts of the automobile such as the steering wheel, dashboard, window, or seatback depending on occupant location. Potential reasons for the vulnerability of females to other causes of concussion such as certain sports include less well-developed neck muscles in females^19^ and lower effective mass of the head during an impact.^20^ Thus, it is surprising that female anthropomorphic test devices (“crash test dummies”) are generally not placed in the driver’s seat for most MVC tests including rear-end collisions. Furthermore, in the past, only small-size (fifth percentile) female anthropometric test forms were typically employed.^4^ Also, in rear-end collisions, the Insurance Institute of Highway Safety (IIHS) had confined its testing to seat design, although recently it began testing other features of rear-end collisions such as the seat restraint.^21^ The National Highway Safety Administration (NHTSA) rear-end collision testing to date has been restricted to studying the integrity of the fuel system.^22^

Specially lacking is the availability of anthropometrically appropriate female forms for the full range of female sizes. Although this deficiency has recently been partially corrected with the Swedish introduction of appropriate female test forms labeled VIVA+ forms made to represent both the 50% male and 50% female forms, the latter being previously unavailable. Recently, these forms were used to assess rear impacts, and it is of interest that the risk of whiplash with the average female form was 1.5 times higher compared to the average male form.^23^ Thus, when appropriate female forms were used for whiplash studies, females were significantly less protected than males. In our view, it is highly likely that this same susceptibility applies to brain injuries such as concussion since the mechanisms of whiplash injury and concussion are very similar. However, the VIVA+ form is only useful for virtual computer simulation, and not for sledge-type studies with actual motor vehicles, and the new human body forms representing the average human male and female show increased susceptibility of females for some types of injury, but concussion has not been studied.

Only 3 of the 136 participants in our study reported complete recovery with an average follow-up duration of 30 months (IQR 16.8–58.5), and those with incomplete recovery suffered prolonged symptom duration. These results are similar to our other studies that showed that concussions sustained in MVCs are associated with worse outcome compared to other causes of concussion.^2,14^ Some potential explanations suggested by others include the greater forces applied to the head in MVCs compared to other causes of concussion,^24^ additional psychological stress in MVCs, the presence of additional injuries such as whiplash, and the overlap between PCS and posttraumatic stress.^25^ We have previously shown the high incidence of depression, anxiety, and diminished quality of life after concussions of all causes that can indeed prolong recovery.^26^ There have also been many other theories purporting to explain the worse outcomes in MVC occupant concussions including exaggeration, secondary gain, hysteria, stress, somatization, and fear avoidance, which allegedly contribute to failure to recover from concussion, especially when insurance claims and compensation are involved.^27–29^

An additional issue that may account for failure to recover from C+PCS after MVC or other causes is the delay in recognition of concussion due to the frequent concurrence of both concussion and other injuries, especially whiplash. Indeed, previously whiplash received greater attention than concussion, and older literature on whiplash did not even consider differentiating between whiplash and concussion.^30^ Fortunately, there is now improved recognition of both conditions and greater awareness that they frequently coexist first evidenced in sports concussions^31^ because both injuries are caused by the same linear and rotational acceleration and deceleration forces. Indeed, Table 4 shows their coexistence in MVC in more than two-thirds (68.6%) of the 36 cases with C+PSC who responded to our final questionnaire. Furthermore, Elkin et al.^32^ who studied rear-end collisions to assess the brain strain based on a finite element model of the human brain found evidence of “a potential biomechanical link between whiplash and concussion.” Indeed, there was evidence that when the seat configuration failed to restrain the head after a rear-end MVC, the patient could sustain a mechanical insult to the brain “typically associated with sports-related head impacts.” It is of interest that Jakobsson et al.^16^ showed that rear-end collisions have the highest incidence of whiplash that was also true in the present study. Previous prevention efforts were often focused on seatbelts and seat design including head restraints to prevent whiplash and severe brain injuries. Unfortunately, these prevention strategies have been insufficient for preventing concussion because many patients in the present study had both concussion of the brain and whiplash of the neck and were wearing seatbelts and had the head restraint in the prescribed location (Tables 1, 2, and 4). These measures were insufficient to prevent the bobble-head effect.

## Conclusions

Our study shows that current occupant safety measures including seatbelts, airbag location, and indications for deployment, and head restraints are currently insufficient to prevent concussions in MVC. There is an urgent need to consider additional strategies to prevent occupant concussions in MVC, especially after rear-end collisions in women. Other protective mechanisms specific for rear-end collisions should be considered for all occupants no matter where seated and include additional airbag placement and deployment indications and additional cabin padding. It is likely that the enhanced head protection required to prevent brain concussion and neck whiplash will also enhance protection against more severe brain and spinal injuries among occupants in MVC crashes. In contrast to the problems of concussions among occupants of MVC on streets and highways, it is of interest to consider the success of the automobile racing community in engineering race car cockpits and driver harnesses to protect its drivers from head and neck injuries due to the bobble-head phenomenon in all types of collisions. The Hubbard Head and Neck Support System device and surround cockpit protection have been remarkably successful in immobilizing the driver’s head on the trunk and preventing it from moving in any direction after a crash thus saving lives and preventing brain and cervical spine injuries in motor racing sports.^33^ Of course, this system is impractical for MVC occupants on roads and highways, but the principle of preventing bobble-head due to differential movement of the head on the trunk has been tested and proven and should stimulate efforts to protect the general driving public.

It is of interest to note the similarly higher susceptibility of women than men to sustain a concussion in certain sports such as basketball and soccer previously identified by Gesell et al.^20^ One of the concussion prevention strategies for women athletes is based on anthropometric sex differences in cervical spine musculature, and the remedy suggested is improved strengthening of the cervical spine muscles by measures such as isometric neck muscle exercises.

The failure of both governmental and insurance industry supported test crash facilities to employ female anthropometric forms of all sizes and seated in all motor vehicle occupant locations must be corrected so that new concussion prevention measures will be relevant to females of all sizes, just as has been done for males in frontal and lateral collisions. Given the results of this study, automobile engineers, automobile manufacturers, and governments should be encouraged to improve vehicle occupant safety systems to prevent concussions with a focus on female occupants in rear-end collisions who are at high risk of concussion.

### Limitations

Our study has several methodological limitations some of which are inherent in concussion research in general. Indeed, in the field of concussion, there is an ongoing effort with many recent attempts to achieve better nosology, diagnosis, classification, treatment, recovery, and prevention of concussion and its consequences.^10,34,35^ In particular, the absence of highly specific and sensitive biomarkers of concussion and C+PCS is a continuing problem hampering prevention efforts. Other limitations of our study is that all the patients with C+PCS were seen by physicians at one tertiary hospital concussion clinic under the supervision of one of the authors (CHT). Furthermore, even though the included patients were a consecutive cohort followed prospectively, they are not representative of the entire spectrum of patients with concussion. For example, by policy, the clinic only sees adults of any age, but youths must be at least 11 years of age, and importantly, our concussion clinic is a referral center specializing in PCS and in those with cognitive decline suspected of chronic traumatic encephalopathy. In general, we do not see acutely concussed patients, especially during the first month after injury during which time about 75% of concussed patients recover. Another potential limitation of the study relates to the narrative system of symptom recording by physicians specializing in concussion versus the use of one of the many self-report symptom checklists such as SCAT3. There is limited evidence for which system is more accurate. For example, Meier et al.^36^ studied self-reporting of concussion symptoms and found that athletes reported significantly fewer symptoms using standardized self-report symptom measurements compared to reporting during confidential in-person consultations. Also, there is recall bias with a study of this duration where subjects are asked to answer questions about events that occurred many years ago.

Our recently published study of the causes of concussion in 600 patients, MVC accounted for 100 of the cases.^2^ In that study, we used a less liberal definition of PCS consisting of at least three symptoms lasting for at least one month and assessed the relationship between the cause of concussion and the number of symptoms. We found that concussions caused by MVC produced a median number of 11 concussion symptoms per patient, which was significantly higher than all the other causes of concussion (Table 3). In contrast, in the current study of 136 patients with MVC occupant concussions, the inclusion criterion was only one persisting symptom, but we found that the total symptoms per patient averaged 13.39 (Table 2). Thus, the two studies encompassed not only different accrual dates but also differed in the number of required persisting symptoms. However, in the present study, only four patients had less than three symptoms (one patient had only one symptom, and three patients had only 2 symptoms). The comparison of results between the two studies is a type of “surrogate sensitivity analysis” of the current article because different numbers of persisting symptoms were used. Both studies showed that patients with MVC suffered from multiple symptoms, but concussed patients accepted for the study with the more liberal definition of PCS were more symptomatic.

Another potential limitation is the mainly retrospective nature of this study, except for the final follow-up MVC-focused questionnaire in which the response rate was low, but we did gather some data on preexisting comorbidities. However, we have no reason to believe that such comorbidities would differ by type of collision, and therefore, it is unlikely they would confound the association of type of MVC with the number of persistent symptoms. Also, the study population involved only concussed occupants with PCS and did not include a control group of uninjured or concussed but recovered occupants without PCS. A detailed reconstruction of each collision including examination of the crashed vehicles would likely have identified additional prevention strategies, such as distance warning, but this is outside the scope of the present study. With respect to recovery, we were not able to assess the extent of recovery in many with incomplete recovery. Since so few patients recovered completely, there was limited ability to compare the completely recovered patients to those with incomplete recovery.

## Transparency, Rigor, and Reproducibility

The study was approved by the University Health Network Research Ethics Board (13-6167). The study and analysis plan were not formally preregistered because the decision to perform this specific analysis of MVC C+PCS arose during the analysis of our recently published study of the types of causes of C+PCS^2^ rather than at the beginning of patient accrual. Also, this was not a control trial, but rather a longitudinal, retrospective, and prospective study. Therefore, we concluded that Research Ethics Board approval was sufficient. No power analysis was performed for this study. A total of 815 patient charts were reviewed to determine eligibility for the study, and 136 patients met the inclusion criteria. Recovery was assessed based on clinic visits and returned questionnaires. Patients with known incomplete recovery attended at least one clinic visit or returned at least one questionnaire, in which they did not report complete recovery, and thus they were not available for further follow-up. Patients with unknown recovery had incomplete assessments because they failed to attend a follow-up clinic visit or return a questionnaire and did not report complete recovery at their only visit. Data were collected by trained research assistants through chart review of patients who came for a first initial clinic visit between the years 2000 and 2020 and from follow-up clinic visits or returned questionnaires. Research assistants could not be blinded because they needed to extract all the information from patient records. All questionnaires used in the study are available if requested from the authors. All information collected was confidential, and patients had a unique study ID so that patients’ names were not associated with any information collected. The methods section explains the nature of the statistical tests used. Two of the co-authors (Q.L. and E.H.) are statisticians and performed all the analyses using R.4.2 (R Foundation for Statistical Computing, Vienna, Austria). All authors have agreed to publish the article using the Mary Ann Liebert. Inc “Open Access” option, and with the appropriate license it will be freely available upon publication.
